# Real-world evidence for atezolizumab with bevacizumab for unresectable hepatocellular carcinoma: a multicentre UK Midlands cohort

**DOI:** 10.1186/s12885-026-16387-3

**Published:** 2026-07-20

**Authors:** Richard Phillips, Harry Daniels, Marie Quinlan, Ali Suwaidan, Kaushal Maru, Jake Hancock, Mabel Teng, Jasvinder Kaur, Yuk Ting Ma, Sean Dulloo, Sadaqat Hussain, Basma Greef, Shivan Sivakumar

**Affiliations:** 1 Department of Immunology and Immunotherapy, School of Infection, Inflammation and Immunology, College of Medicine and Health, University of Birmingham, Birmingham, B15 2TT UK; 2 Department of Medical Oncology, University Hospitals Birmingham NHS Trust, Edgbaston, Birmingham, UK; 3 Department of Oncology and Radiotherapy, Nottingham University Hospitals NHS Trust, Nottingham, UK; 4 Department of Medical Oncology, University Hospitals Leicester, Leicester, UK; 5 Divisional Research Lead for Oncology, Royal Surrey Cancer Centre, GU2 7XX, Surrey, UK

**Keywords:** HCC, Immunotherapy, Real-world evidence, Atezolizumab-bevacizumab, Immune-related adverse events

## Abstract

**Background:**

The IMbrave150 trial established atezolizumab plus bevacizumab (ATE/BEV) as first-line standard of care for unresectable hepatocellular carcinoma (HCC), demonstrating superior overall survival (OS) and progression-free survival (PFS) versus sorafenib. Real-world data are required to further evaluate effectiveness and safety.

**Methods:**

We conducted a retrospective multicentre analysis across three oncology centres in the Midlands, UK, with audit committee approval. Eligible patients had HCC confirmed histologically where feasible.

**Results:**

As of August 2025, 233 patients were included, with a median follow-up of 17.4 months (95% CI: 12.6–23.2). Median OS was 16.5 months (95% CI: 14.2–20.7), with 6-month OS of 76.8%. Median PFS was 12.3 months (95% CI: 10.0–16.3), with 6-month PFS of 70.8%. The objective response rate was 42.9%. Treatment discontinuation occurred in 81.5% of patients, most commonly due to disease progression (47.4%), toxicity (22.6%), or liver decompensation (17.9%). Second-line therapy was administered to 39.1%. Any-grade adverse events occurred in 41.2%, with ≥grade 3 toxicity in 23.2%.

**Conclusions:**

In this UK real-world cohort, ATE/BEV demonstrated survival outcomes comparable to IMBrave150, with manageable toxicity and meaningful access to subsequent therapy, supporting its effectiveness beyond clinical trial settings.

**Graphical Abstract:**

**Figure:**
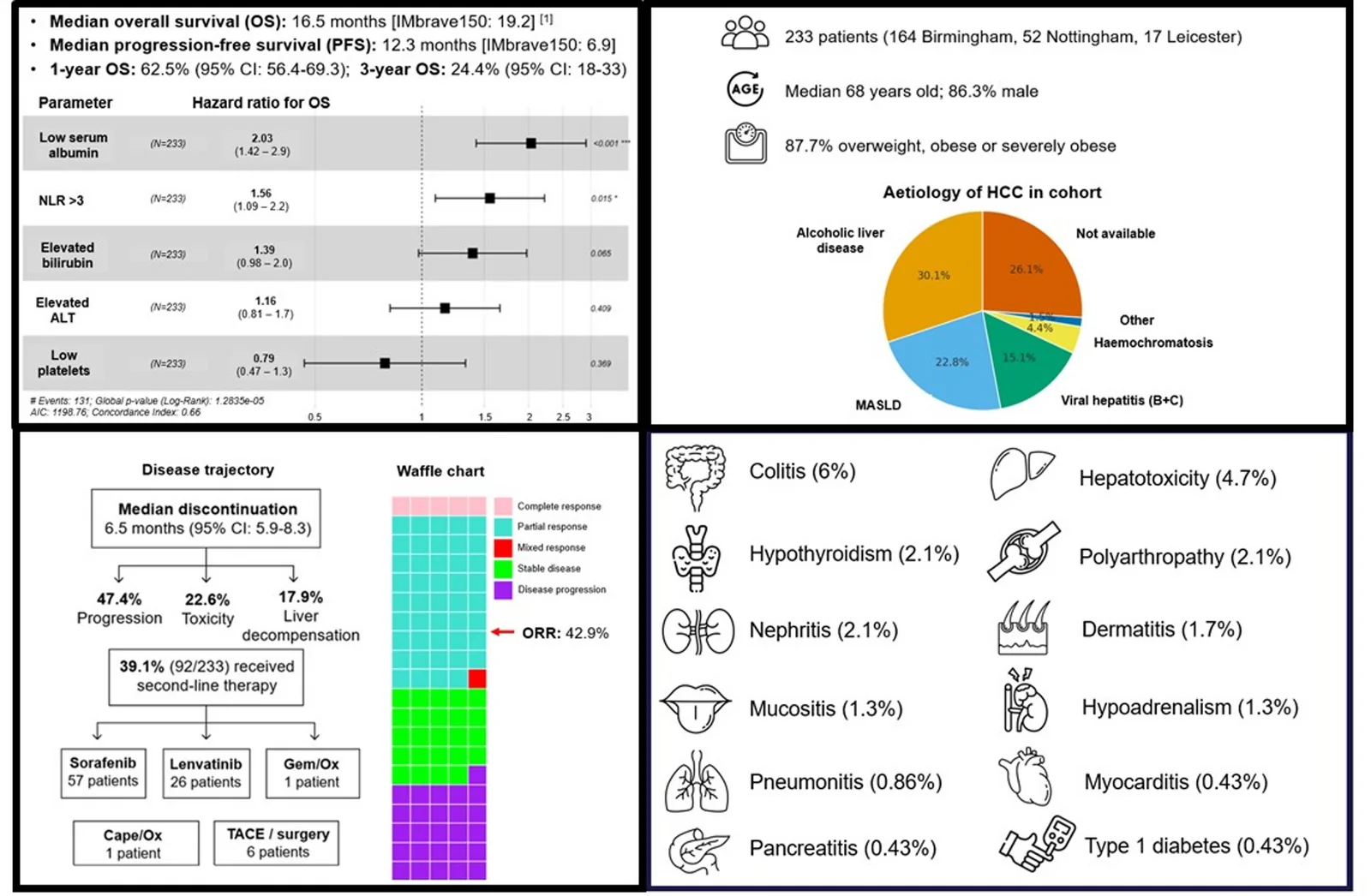

## Introduction

Hepatocellular carcinoma (HCC) is the most common primary liver malignancy, accounting for approximately 80–90% of all liver cancers [1, 2]. Globally, HCC is the third leading cause of cancer-related death, with an estimated 830,000 deaths annually and a 5-year relative survival rate of about 18% [1, 3]. In the United Kingdom, liver cancer incidence and mortality have risen sharply over the past two decades, mirroring trends seen across Europe and North America [4–6].

The pathogenesis of HCC is multifactorial, with most cases arising in the context of chronic liver disease and cirrhosis. Major aetiologies include chronic hepatitis B virus (HBV) and hepatitis C virus (HCV) infection, alcohol-related liver disease, and increasingly, metabolic-associated steatotic liver disease (MASLD) and metabolic-associated steatohepatitis (MASH) [1, 2, 4]. Metabolic risk factors, such as obesity and diabetes are contributing to the rising incidence in Western countries, where non-viral causes now predominate [4]. Other less common causes include hereditary haemochromatosis, aflatoxin exposure (primarily in Asia), and rare metabolic disorders [1].

The global burden of HCC is expected to increase by more than 50% over the next 20 years, driven by demographic shifts, persistent viral hepatitis, and the epidemic of metabolic liver disease [4]. In the UK, the age-standardised incidence rate for liver cancer is approximately 9 per 100,000, with mortality closely paralleling incidence due to poor prognosis and limited curative options for advanced disease [6]. The rising prevalence underscores the need for improved prevention, early detection, and effective systemic therapies [7].

Atezolizumab plus bevacizumab (ATE/BEV) represents an immunotherapy-based regimen that has demonstrated efficacy in the first-line treatment of unresectable HCC. This regimen was evaluated in a pivotal phase III trial, IMbrave150, and compared against the standard of care tyrosine kinase inhibitor, sorafenib [8]. The IMbrave150 trial demonstrated that ATE/BEV significantly improved both overall survival (OS) and progression-free survival (PFS) compared to sorafenib in treatment-naive patients [8]. With a median follow-up of 15.6 months, the median OS for ATE/BEV was 19.2 months versus 13.4 months for sorafenib (HR 0.66; *p* < 0.001), and median PFS was 6.9 vs. 4.3 months (HR 0.65; *p* < 0.001).

In IMbrave150, the combination was associated with hypertension, proteinuria, and bleeding from bevacizumab, and immune-mediated hepatitis and colitis from atezolizumab. The combination was generally well tolerated, with grade 3/4 treatment-related adverse events occurring in 43% of patients. This trial established ATE/BEV as a new standard of care. Given the high prevalence of cirrhosis in HCC, the tolerability of these agents remains a critical question.

Studies from Asia and North America have reported on the real-world effectiveness and safety of this treatment regimen [9–12]. UK practice differs from previously reported cohorts with respect to liver disease aetiology, access to endoscopic surveillance, and thresholds for treatment continuation, warranting region-specific real-world evaluation.

This report of ATE/BEV in patients with unresectable HCC treated across three oncology centres in the UK Midlands evaluates survival, safety, and treatment pattern. Understanding how this regimen performs in routine practice will inform optimal first-line treatment selection and guide clinical decision-making as both therapies become more widely accessible.

## Materials and methods

### Study population

The study population comprised patients with advanced hepatocellular carcinoma (HCC), defined as either locally advanced or metastatic disease. Eligible patients were required to have a diagnosis of HCC confirmed by histological biopsy. Patients received first-line palliative treatment with atezolizumab in combination with bevacizumab. Individuals who commenced first-line therapy with tyrosine kinase inhibitors, such as lenvatinib or sorafenib, were excluded from analysis.

Clinical data were retrospectively collected from three oncology centres in the United Kingdom. Approval for the study was obtained from the local audit committees at each participating centre. Patients with incomplete follow-up were censored at last contact.

A cutoff of 24.2 ng/ml (upper limit of normal) was used for an elevated alpha fetoprotein (AFP).

### Statistical analysis

The primary endpoint was overall survival (OS), defined as the time from initiation of atezolizumab plus bevacizumab to death from any cause or last follow-up. Secondary endpoints included progression-free survival (PFS), objective response rate (ORR), disease control rate (DCR), duration of response (DoR), safety outcomes, and the impact of categorical variables on survival outcomes. Progression-free survival (PFS) was defined as the interval from treatment initiation to radiological or clinically presumed disease progression, treatment discontinuation due to progression, or death. Clinically presumed progression without radiological confirmation occurred in a small minority of cases (< 20 patients). ORR was defined as the proportion of patients achieving a complete response (CR) or partial response (PR). DCR was defined as the proportion of patients achieving a CR, PR or stable disease (SD). DoR was defined as time from first documented response in those with CR or PR to the point of progression.

Radiological assessment was performed using computed tomography (CT) or magnetic resonance imaging (MRI) approximately every 12 weeks in accordance with routine clinical practice across participating centres. Imaging intervals were not strictly protocol-mandated, and minor variability occurred where patients underwent imaging locally with subsequent central review at the treating oncology centre. Tumour response was assessed using RECIST version 1.1 criteria across all centres, as was the criteria used in the original IMBrave150 trial. Adverse events (AEs) were retrospectively identified through review of electronic medical records, clinic correspondence, and laboratory results. Toxicities were graded according to Common Terminology Criteria for Adverse Events (CTCAE) version 5.0 where sufficient clinical information was available. Immune-related and bevacizumab-associated toxicities were recorded based on treating clinician attribution. Liver decompensation events were classified as treatment-related only where a clear temporal and clinical association with therapy was documented; otherwise, these events were considered attributable to underlying liver disease or tumour progression. Variability in clinical documentation and reporting between centres, and within centres, may have led to inconsistencies in data capture, and may represent a potential source of information bias.

Survival distributions were estimated using the Kaplan–Meier method, and differences between groups were assessed with the log-rank test. Multivariable analysis was conducted using the Cox regression, with serum albumin, alanine transaminase (ALT), platelets and bilirubin, and neutrophil: lymphocyte ratio (NLR) within 7 days of initiating treatment. Serum albumin was dichotomised at 35 g/L (< 35 versus ≥ 35 g/L), bilirubin at 17 µmol/L (< 17 versus ≥ 17 µmol/L), platelet count at 150 × 10⁹/L (< 150 versus ≥ 150 × 10⁹/L), ALT at 55 U/L (< 55 versus ≥ 55 U/L), and NLR at 3 (≤ 3 versus > 3) for inclusion in multivariable Cox regression analyses.

NLR was selected on the basis of it being implicated as a potential biomarker predicting survival in HCC treated with atezolizumab/bevacizumab [13, 14]. Albumin was selected due to previous analysis of the IMBrave150 trials [15]. Bilirubin, ALT and platelet levels were used a routinely tested surrogate for liver function. Missing data was omitted from multivariable analysis, reducing statistical power and increasing the width of the confidence interval for the particular covariable. Missing data proportion was ≤ 10% for all model variables except aetiology. Associations between categorical variables were analysed using Fisher’s exact test. Statistical significance was defined as a two-sided p-value < 0.05. Analyses were conducted in R version 4.3.1 (R Foundation for Statistical Computing, Vienna, Austria) using the *survival* package for survival analyses and *ggplot2* for graphical visualisation.

## Results

### Demographics

Clinical data were retrospectively collected from three oncology centres in the Midlands, United Kingdom, with 164 patients from Birmingham, 52 from Nottingham, and 17 from Leicester. Baseline demographic and clinical characteristics of the study population (233 patients) are summarised in Table 1. Notably, aetiology was not clarified within clinical documentation in 71 cases.

**Table 1:** Baseline demographics and clinical characteristics of the study population

Demographic parameter	Median (IQR)
Age	68 (63–74)
	**N (%)**
Sex	
Male	201 (86.3%)
Female	32 (13.7%)
Ethnicity	
White	182 (78.1%)
African	12 (5.15%)
Asian	16 (6.87%)
Not available	23 (9.87%)
BMI grouping (ethnicity adjusted)	
Healthy weight	52 (22.3%)
Overweight	92 (39.5%)
Obese	79 (33.9%)
Severely obese	10 (4.29%)
Metastatic	168 (72.1%)
Aetiology	
Alcoholic liver disease	82 (35.15%)
Metabolic-associated steatotic liver disease (MASLD)	62 (26.6%)
Viral hepatitis C	27 (11.6%)
Viral hepatitis B	14 (6.01%)
Haemochromatosis	12 (5.15%)
Primary biliary cholangitis	2 (0.86%)
Alagille syndrome	1 (0.43%)
Fibrolamellar hepatocellular carcinoma	1 (0.43%)
Not available	71 (30.43%)
Diabetic status	
Yes	92 (39.5%)
No	141 (60.5%)
Smoking status	
Current or ex-smoker	116 (49.8%)
Non-smoker	110 (47.23%)
Not available	7 (3%)
Alcohol (weekly intake)	
< 14 units	165 (70.8%)
14–28 units	34 (14.6%)
29–42 units	8 (3.43%)
> 42 units	26 (11.2%)
Baseline alpha fetoprotein (AFP)	
Elevated	140 (60.1%)
Normal	93 (39.9%)
Neutrophil : lymphocyte ratio (NLR)	
< 3	108 (46.4%)
> 3	125 (53.6%)
Liver function (Child-Pugh score)	
A5	113 (48.5%)
A6	68 (29.2%)
B7	20 (8.5%)
B8	6 (2.6%)
Not available	26 (11.2%)
ECOG performance status	
0	62 (26.6%)
1	150 (64.4%)
Not available	21 (9.0%)
BCLC Staging	
B	41 (17.6%)
C	160 (68.7%)
Not available	32 (13.7%)
Tumour burden	
Macrovascular invasion (e.g. portal vein)	47 (20.2%)
Extrahepatic spread	60 (25.8%)
Both	9 (3.9%)
Not available	26 (11.5%)
Prior locoregional therapies	
Transarterial chemoembolisation (TACE)	82 (35.2%)
Stereotactic ablative radiotherapy (SABR)	1 (0.4%)
Both	14 (6.0%)
Not available	26 (11.5%)

### Overall survival and progression-free survival

**Fig. 1:**
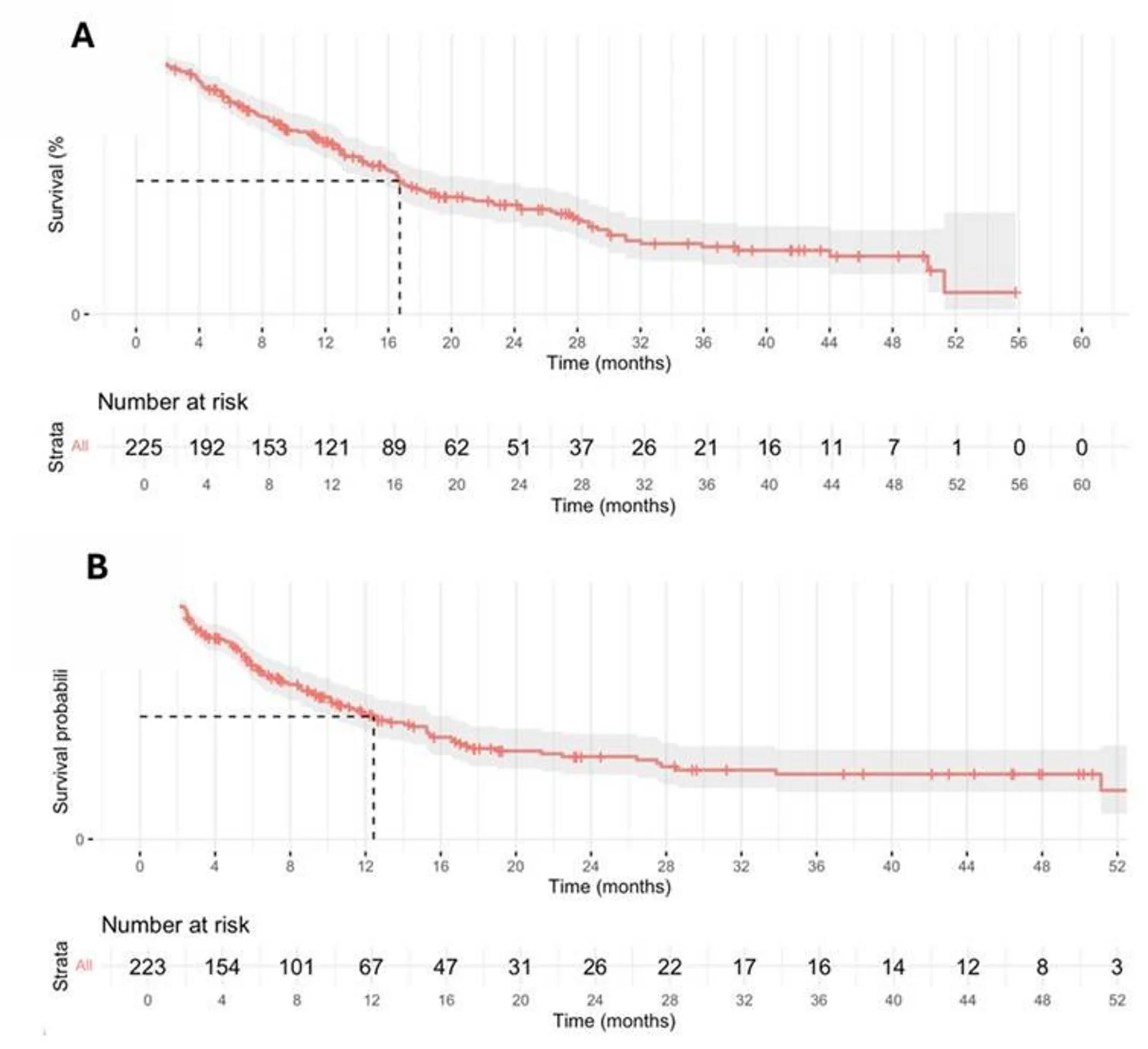
**A** Kaplan-Meier curve demonstrating the overall survival of the population of interest; **B** Kaplan-Meier curve demonstrating the progression-free survival of the population of interest

As of August 2025, median time to follow-up was 17.4 months, with censoring applied for patients alive at last follow-up [95% CI: 12.6–23.2]. 142 patients (60.94%) had died. The median overall survival was 16.5 months [95% CI: 14.2–20.7], with a 6-month overall survival of 76.8% [95% CI: 71.6–82.5] (Fig. 1A). The median progression-free survival was 12.3 months [95% CI: 10–16.3], with a 6-month progression-free survival of 70.8% (95% CI: 64.6–77.6) (Fig. 1B).

In this cohort of 233 patients, there was no statistically significant difference in survival by ethnicity, sex, BMI grouping or aetiology. Similarly, the absence of diabetes mellitus, positive smoking status or excessive alcohol intake did not confer any survival difference.

**Fig. 2:**
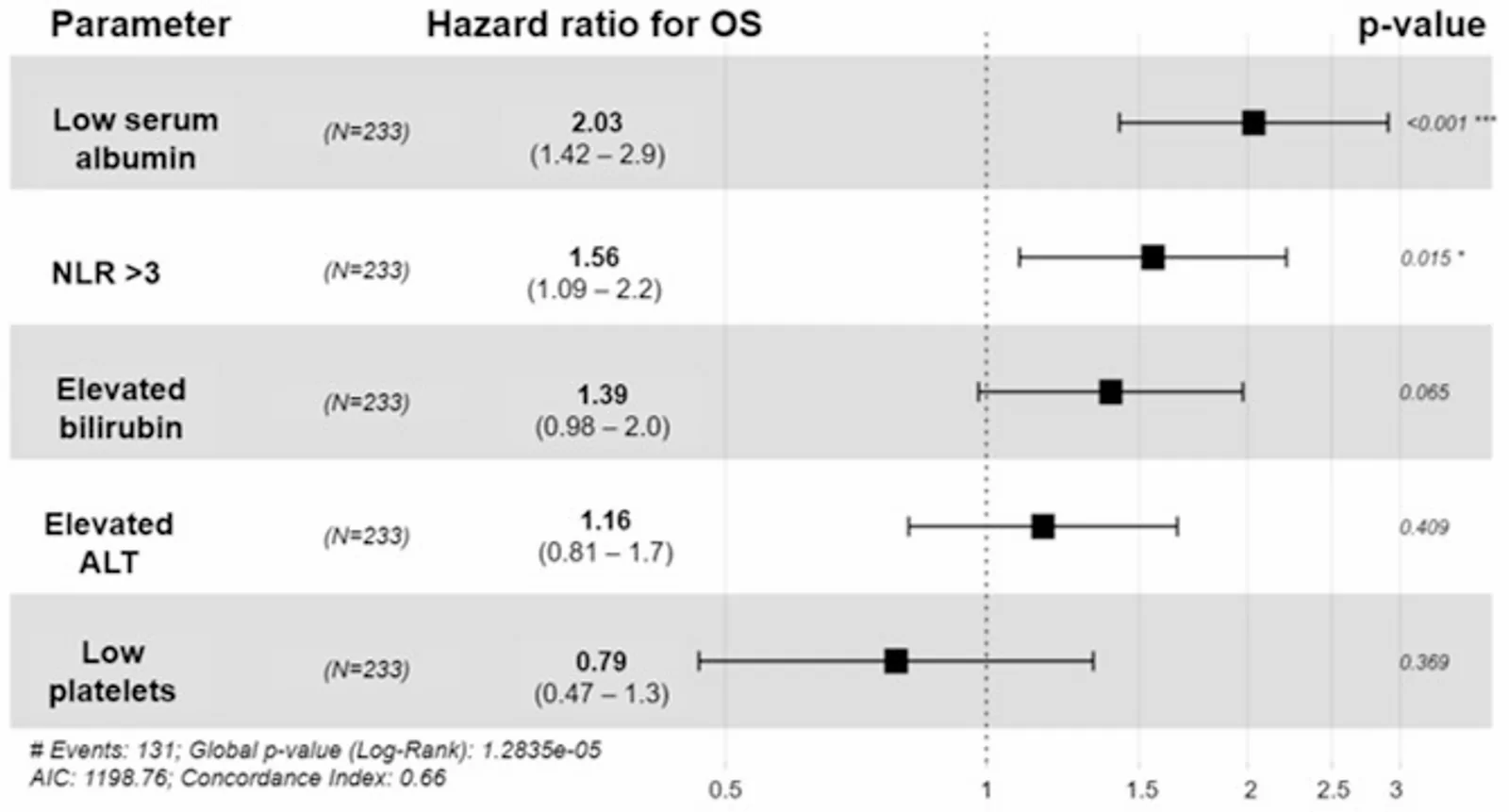
Forest plot demonstrating hazard ratios for association between overall survival and key biochemical parameters. Serum albumin was dichotomised at 35 g/L (< 35 versus ≥ 35 g/L), bilirubin at 17 µmol/L (< 17 versus ≥ 17 µmol/L), platelet count at 150 × 10⁹/L (< 150 versus ≥ 150 × 10⁹/L), ALT at 55 U/L (< 55 versus ≥ 55 U/L), and NLR at 3 (≤ 3 versus > 3) for inclusion in multivariable Cox regression analyses

Using multivariable Cox regression, the overall survival hazard was correlated against key biochemical parameters: serum albumin, NLR, bilirubin, ALT and platelets. Low serum albumin and NLR > 3 were both significantly associated with poorer overall survival (Fig. 2). Raised ALT and bilirubin, and low platelets were not associated with survival.

### Response rates, cycle maintenance and second-line therapies

**Fig. 3:**
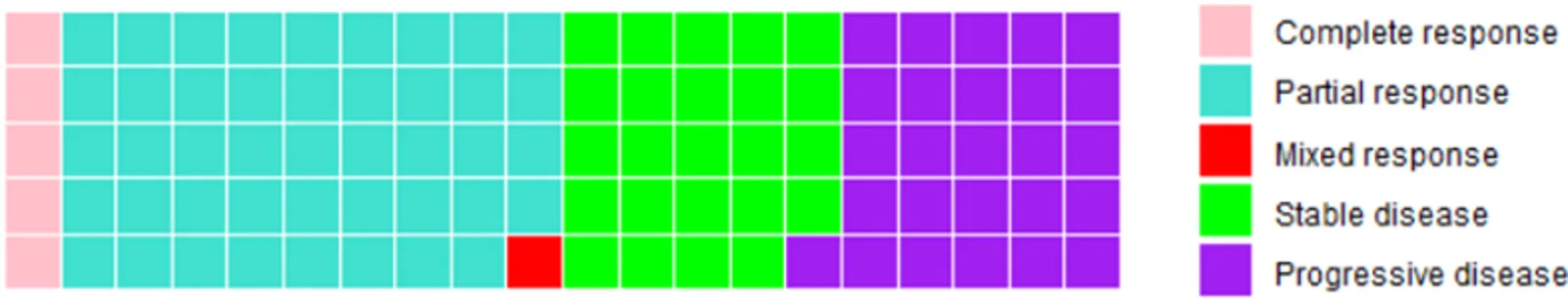
Waffle chart illustrating the proportion of response seen on treatment with atezolizumab with bevacizumab. Complete response (CR), *n* = 3. Partial response (PR), *n* = 97. Mixed response, *n* = 2. Stable disease (SD), *n* = 60. Progressive disease (PD), *n* = 71

The ORR was 42.9%, with 100 patients achieving a partial (PR) or complete response (CR). The proportional representation is demonstrated in the waffle chart in Fig. 3. The DOR was 217 days (IQR 89–453). The DCR was 68.7%, with 160 patients achieving stable disease (SD), PR or CR.

A median number of 8.32 cycles of treatment were offered, with interquartile range of 10. 190 of 233 (81.54%) patients discontinued the treatment at time of analysis, with a median time to discontinuation of 6.5 months (95% CI: 5.9–8.3 months). Reasons for discontinuation included progression (47.4%), toxicity (22.6%) and liver decompensation (17.9%). 91 of 233 (39.06%) patients received a second-line therapy after stopping atezolizumab with bevacizumab. Subsequent therapies are highlighted in Table 2.

**Table 2:** Second-line treatment options provided to patients who discontinued first-line treatment, *n* = 91

Second-line option	*N* (%)
Sorafenib	57 (62.6%)
Lenvatinib	26 (28.6%)
DEB-TACE	3 (3.3%)
Gemcitabine-oxaliplatin	2 (2.2%)
Capecitabine-oxaliplatin	1 (1.1%)
Liver resection	1 (1.1%)
Metastatectomy	1 (1.1%)

### Safety profile

A total of 96 patients experienced adverse events (41.2%) of any grade, with 54 patients (23.2%) experiencing a grade 3 toxicity or above that warranted treatment cessation of one or both agents. A total of 74 patients (31.8%) experienced atezolizumab-related adverse event, all grade 2 or above. A total of 26 (11.2%) patients experienced a bevacizumab-related toxicity of grade 2 or above. 24 of the evaluable patients were on atezolizumab monotherapy due to baseline contraindications to bevacizumab (e.g. untreated varices).

The spectrum of adverse events was diverse and shown in Table 3.

**Table 3:** Summary of atezolizumab and bevacizumab-related adverse events experienced by the population of interest

Atezolizumab-related toxicity	Number (%)	Bevacizumab-related toxicity	Number (%)
Hepatotoxicity	11 (4.7%)	Thrombocytopaenia	3 (1.3%)
Colitis	14 (6%)	Proteinuria	18 (7.7%)
Hypothyroidism	*5 (2.1%)*	*Hypertension*	*2 (0.86%)*
Polyarthropathy	*5 (2.1%)*	*Gastrointestinal bleed*	*1 (0.43%)*
Nephritis	*5 (2.1%)*	*Haemoptysis*	*1 (0.43%*
Dermatitis	*4 (1.7%)*	*Renal dysfunction*	*1 (0.43%)*
Mucositis	*3 (1.3%)*	*Anaemia*	*1 (0.43%)*
Hypoadrenalism	*3 (1.3%)*		
Pneumonitis	*2 (0.86%)*		
Fatigue	*2 (0.86%)*		
Bullous pemphigoid	*1 (0.43%)*		
Cardiomyopathy	*1 (0.43%*		
Guillain-Barre syndrome	*1 (0.43%)*		
Myocarditis	*1 (0.43%)*		
Pancreatitis	*1 (0.43%)*		
Type 1 diabetes mellitus	*1 (0.43%)*		
Stevens-Johnson syndrome	*1 (0.43%)*		
Xerostomia	*1 (0.43%)*		
Behcet’s-like mucositis	*1 (0.43%)*		

## Discussion

Since the publication of the IMbrave150 trial, atezolizumab plus bevacizumab (ATE/BEV) has been widely adopted as the first-line standard of care for patients with unresectable hepatocellular carcinoma (HCC) [8]. Several real-world studies from Asia, Europe and North America have since validated its clinical efficacy and tolerability outside the confines of trial populations [9–12]. The present multicentre UK analysis contributes to this growing body of evidence by describing outcomes in a cohort of 233 patients treated across three oncology centres in the Midlands, reflecting pragmatic clinical practice in a diverse patient population.

In this study, the median overall survival (OS) was 16.5 months, findings broadly consistent with those observed in IMbrave150 (median OS 19.2 months). The slightly shorter survival in our cohort likely reflects differences in baseline characteristics, including a higher burden of comorbidity and a greater prevalence of alcohol-related liver disease. The median progression-free survival (PFS) was 12.3 months, numerically longer than that reported in IMbrave150 (6.9 months). This difference should be interpreted cautiously, as radiological assessments in our cohort were performed approximately every 12 weeks in accordance with routine clinical practice rather than under strictly protocol-mandated trial conditions. Variability in imaging intervals and the small proportion of clinically presumed progression events may have introduced interval-censoring, whereby the estimated progression date does not accurately reflect the biological reality, rather the point at which the progression was detected.

The objective response rate (ORR) of 42.9% aligns with IMbrave150 (30%). This high response rate translates into meaningful clinical benefit, particularly in patients with preserved liver function. Importantly, 39% of our patients received subsequent systemic therapy following ATE/BEV discontinuation, reflecting an evolving treatment paradigm in which sequential tyrosine kinase inhibitors are increasingly feasible in real-world practice.

Biochemical and inflammatory markers demonstrated prognostic significance in this cohort. Low serum albumin (< 35 g/L) was independently associated with increased mortality risk (HR 2.03), consistent with the established prognostic importance of preserved hepatic synthetic function and with established prognostic models such as the ALBI score [16]. The neutrophil-to-lymphocyte ratio (NLR) > 3 was predictive of poorer outcomes (HR 1.56), corroborating existing literature identifying systemic inflammation as a biomarker of impaired immunotherapeutic response [17]. These findings support the integration of simple, reproducible biomarkers such as NLR or serum albumin into future prognostic stratification and patient selection strategies.

Adverse events of grade ≥ 3 occurred in 23.2% of patients, consistent with IMbrave150 (43%) and subsequent observational series (range 20–30%). Immune-related toxicities attributable to atezolizumab occurred in 31.8% of patients, most commonly hepatitis and colitis, while bevacizumab-related adverse events (11.2%) were dominated by hypertension, proteinuria and gastrointestinal bleeding. Notably, the rate of bleeding events was lower than that reported in Asian cohorts, which may reflect careful endoscopic screening and prophylaxis for varices prior to initiation in UK centres, or decision to treat with atezolizumab monotherapy. Notably, the incidence of colitis was numerically higher in our cohort (6%) than reported in IMbrave150 (1–2%) and may reflect differences in real-world reporting practices, diagnostic thresholds, or case ascertainment. Overall, ATE/BEV toxicity was manageable, with most events reversible upon cessation or corticosteroid therapy, reaffirming its tolerability in cirrhotic patients when delivered under multidisciplinary oversight.

The retrospective design of this study inevitably introduces limitations. Absence of central radiology review may lead to under- or overestimation of progression-free survival, and median follow-up duration remains relatively short at 17.4 months. Inter-centre variability in treatment continuation and imaging schedules may also have contributed to heterogeneity in survival estimates. Although imaging was generally performed at approximately 12-week intervals, assessments reflected routine clinical practice rather than strictly protocolised trial schedules, and absence of central radiology review may have contributed to overestimation of progression-free survival. Retrospective toxicity assessment may have resulted in under-reporting of lower-grade adverse events that were not consistently documented in routine clinical records, although clinically significant grade ≥ 3 toxicities and hospitalisation-associated events were more reliably captured.

Furthermore, the study did not include a comparator arm, precluding direct assessment of relative efficacy versus other treatments options. Examples would include lenvatinib or the newer first-line immunotherapy STRIDE regimen (durvalumab plus tremelimumab) [18]. Another limitation is that baseline HCC aetiology was unavailable for approximately 30% of patients. As underlying liver disease may influence prognosis, liver function trajectories, and potentially response to systemic therapy, incomplete aetiological data may have limited our ability to fully characterise the cohort and evaluate outcome differences between aetiological subgroups.

Despite these constraints, the sample size and multicentre representation lend robustness to the findings, and the results are consistent with international RWE datasets.

Looking ahead, the therapeutic landscape of advanced HCC continues to evolve. Real-world comparisons between ATE/BEV and STRIDE are anticipated, particularly as both regimens demonstrate similar survival outcomes but differing safety profiles. Biomarker-driven selection and adaptive sequencing strategies may further refine treatment choice. Future prospective registry data integrating genomic and immunologic profiling will be instrumental in delineating predictors of long-term benefit and in identifying patients who may achieve durable remission or treatment-free survival.

In summary, this UK Midlands real-world cohort demonstrates that atezolizumab plus bevacizumab achieves survival and response outcomes comparable to clinical trial data, with manageable toxicity and preserved access to subsequent therapy. These findings confirm the effectiveness of ATE/BEV in routine clinical practice and support its continued role as the preferred first-line option for unresectable hepatocellular carcinoma.

## Conclusions

Our results are broadly consistent with the IMbrave150 trial and subsequent multicentre RWE studies. There is a benefit to patients for the addition of immunotherapy to chemotherapy in the real-world setting. Ongoing real-world comparisons between ATE/BEV and STRIDE are warranted, given that each regimen has only been compared to sorafenib. However, given their differing safety profile, it is likely that both regimens will occupy a space within the palliative management of advanced hepatocellular carcinoma for the present time.

## Data Availability

The datasets used and/or analysed during the current study are available from the corresponding author on reasonable request.
